# Protective effect of cafestol against doxorubicin-induced cardiotoxicity in rats by activating the Nrf2 pathway

**DOI:** 10.3389/fphar.2023.1206782

**Published:** 2023-06-12

**Authors:** Sara A. Al-Kenany, Nada N. Al-Shawi

**Affiliations:** Department of Pharmacology and Toxicology, College of Pharmacy, University of Baghdad, Baghdad, Iraq

**Keywords:** doxorubicin, cafestol, oxidative stress, Nrf2 pathway, cardiotoxicity

## Abstract

Doxorubicin (DOX) is an efficient antineoplastic agent with a broad antitumor spectrum; however, doxorubicin-associated cardiotoxic adverse effect through oxidative damage and apoptosis limits its clinical application. Cafestol (Caf) is a naturally occurring diterpene in unfiltered coffee with unique antioxidant, antimutagenic, and anti-inflammatory activities by activating the Nrf2 pathway. The present study aimed to investigate the potential chemoprotective effect of cafestol on DOX-induced cardiotoxicity in rats. Wistar albino rats of both sexes were administered cafestol (5 mg/kg/day) for 14 consecutive days by oral gavage alone or with doxorubicin which was injected as a single dose (15 mg/kg intraperitoneally at day 14) to induce toxicity. The result showed that Caf significantly improved cardiac injury induced by doxorubicin, decreased serum levels of CK-MB, LDH, ALP, and ALT, and improved histopathological changes. In addition, cafestol significantly inhibited DOX-induced cardiac oxidative stress as seen in the reduced level of MDA and increased GSH, SOD, CAT, and Gpx-1 cardiac tissue levels; cafestol significantly enhanced Nrf2 gene and protein expression and promoted the expression of downstream antioxidant genes HO-1 and NQO-1 and downregulated Keap1 and NF-κB genes’ expression; in addition, Caf significantly reduced inflammatory mediators, TNF-α, and IL-1β levels and inhibited cardiac apoptosis by modulating Bax and Casp 3 tissue levels and reduced TUNEL-positive cardiomyocytes. In conclusion, the present study confirmed that cafestol improved the cardiotoxic effects induced by doxorubicin through the regulation of apoptosis and oxidative stress response through the Nrf2 pathway; this study suggests that cafestol may serve as a potential adjuvant in chemotherapy to alleviate DOX-induced toxicities.

## 1 Introduction

Doxorubicin (DOX), known as “adriamycin,” is an important member of the anthracycline antibiotics that has a broad antitumor spectrum, where it is used worldwide in the treatment of many human malignant cancers as a component of various chemotherapeutic regimens (Sritharan and Sivalingam, 2021). Antitumor activity of doxorubicin, through direct DNA damage, by interfering with the function of many enzymes necessary for DNA replication (Chen, Tzu, Wu, Yan, Chung, Yu, Hwu, Yeukuang, Cheng, Hsun, Mou, Yuan, 2012) (Nebigil and Laurent, 2018) in addition to oxidative stress and overproduction of free radicals, which deplete antioxidants and increase lipid peroxidation, impacts not only cancerous cells but also normal cells leading to toxicity (Quiles et al., 2002) (Swift et al., 2006) (Sirwi et al., 2022). As such, doxorubicin causes severe cardiotoxicity, which is dose-dependent; often results in systolic cardiac dysfunction, dilated cardiomyopathy, and, in extreme cases, heart failure, which has a substantial negative impact on the patient’s health; and poses a significant hurdle in doxorubicin clinical application (Hitchcock-Bryan et al., 1986) (Karim et al., 2001).

The mechanism that mediates doxorubicin-induced cardiotoxicity is unclear, but it is thought to be associated with oxidative stress, inflammatory cascade disorder, autophagy, and apoptotic cell death, in which increased lipid peroxidation, disrupted Ca^2+^ homeostasis, and mitochondrial damage, might account for the leading cause of myocardial injury (Hajra et al., 2018) (Rocca et al., 2020) (Rawat et al., 2021). On the other hand, the nuclear factor erythroid 2-related factor 2 (Nrf2)/Kelch-like ECH-associated protein 1 (Keap1) antioxidant pathway activation displayed a protective effect against cardiovascular diseases, including hypertension, myocardial ischemia, and reperfusion myocardiopathy injury (Barančík et al., 2016). Therefore, studies have concentrated on investigating the possibility of decreasing doxorubicin-mediated cardiotoxicity through attenuating oxidative stress and inhibiting apoptosis via the endogenous antioxidant pathway activation (Dundar et al., 2016) (Gu et al., 2021). Although dexrazoxane is currently the sole chemoprotective agent to prevent and manage cardiotoxicity induced by doxorubicin, its use carries a substantial risk of inducing hematological abnormalities and myelosuppression (Reichardt et al., 2018) (Dallons et al., 2020).

Cafestol is a natural diterpene extracted from the lipid fraction of the coffee bean, which predominantly exists in unfiltered coffee as a fatty ester (Cruchten et al., 2010); it has been shown to counteract oxidative stress (Lee et al., 2007), has protective activity against genetic abnormalities as it prevents the early mutagenic events (Tao et al., 2008), and has an anti-inflammatory effect (Kim, Jung, and Jeong, 2004). Cafestol’s antioxidant effects are related to its ability to activate the Nrf2/antioxidant response element (ARE) pathway, leading to the induction of the expression of antioxidant proteins and enzymes and inhibiting the expression of the inflammatory mediator (Hao et al., 2018); cafestol showed to inhibit high-glucose-mediated cardiac fibroblast proliferation and collagen synthesis by inhibiting proteasomal degradation of Nrf2 and promote its nuclear translocation, causing HO-1 expression upregulation, reducing ROS production, and inhibiting oxidative stress (LiuChen et al., 2020). Furthermore, cafestol exhibited to possess a protective effect against I/R (ischemia-reperfusion) injury by ameliorating inflammation, apoptosis, and autophagy (Ji et al., 2020). In addition, cafestol reduced inflammatory mediators’ production induced by the cyclic strain in human umbilical vein endothelial cells (HUVECs) through activation of the Nrf2/HO-1 pathway and Sirt1 upregulation (Hao et al., 2018). Cafestol treatment might be a new approach to managing oxidative stress-mediated pathophysiological injury.

Therefore, the present study was designed to investigate cafestol’s possible chemoprotective effect on doxorubicin-induced cardiotoxicity through the modulation of the Nrf2 pathway, inflammatory mediator production, and apoptosis inhibition in Wistar albino rats.

## 2 Materials and methods

### 2.1 Chemicals and materials

Cafestol (purity >98%, CAS no. 81760-48-7) was acquired from Sigma-Aldrich (St. Louis, MO 63103, United States). Doxorubicin (doxorubicin HCl 50 mg powder for injection, batch no. 30495, Khandelwal Labs, India) was purchased from local pharmacies. Polysorbate 20 (Tween-20) was purchased from Sinopharm Chemical Reagent Co., Ltd., China. All solvents and chemicals used were of analytical grade.

### 2.2 Dose selection, preparation, and mode of administration

Doxorubicin was dissolved in 0.9% normal saline, and a single dose of 15 mg/kg body weight was administered intraperitoneally based on its success in inducing cardiotoxicity in Wister rats (Sirwi et al., 2022).

Cafestol was prepared as a suspension using 5% Tween-20 in distilled water (D.W.). It was freshly prepared each day just before treatment and was administered orally by oral gavage as a single dose of 5 mg/kg body weight which was optimized based on the previously shown protective effect (Lee et al., 2007) and our preliminary experiment.

### 2.3 Animal care and experimental design

A total of 32 Wistar albino experimental rats of both sexes aged 6 weeks with an average weight of 150 g were used in this study; animals were acquired and maintained in the College of Pharmacy Experimental Animal House, University of Baghdad, Iraq. The experimental animals were kept under controlled conditions of a light/dark cycle (12 h) at a temperature of 23°C ± 2°C and humidity of 50% ± 5%. They had free access to standard commercial diet purchased from the local market and tap water *ad libitum*. The rats were acclimatized prior to the start of the experiment for 1 week. The study protocol was approved by the Graduate Studies and Ethics Committees of the College of Pharmacy, University of Baghdad.

Experimental animals were randomly assigned into four groups (*n* = 8) as follows:

Group I: each rat was given vehicle only (5% Tween in DDW) orally via oral gavage for 14 consecutive days. Then, a single dose of NaCl (0.9%), 10 mL/kg (Turner et al., 2011), was injected intraperitoneally 1 h after the last vehicle administration on day 14. This group served as the normal (negative control) group.

Group II: each rat was orally given cafestol (5 mg/kg/day) for 14 consecutive days.

Group III: each rat was given vehicle only (5% Tween in DDW) via oral gavage for 14 consecutive days. Then, a single dose of doxorubicin (15 mg/kg) was injected intraperitoneally 1 h after the last vehicle administration on day 14 to serve as the positive control group.

Group IV: each rat received cafestol (5 mg/kg/day) orally for 14 consecutive days, and then, a single dose of doxorubicin (15 mg/kg) was injected intraperitoneally 1 h after the last cafestol treatment on day 14.

### 2.4 Serum collection and tissue preparation

Twenty-four hours after doxorubicin dose administration (i.e., day 15), the animals were anaesthetized, and blood samples were collected from the jugular vein in non-heparinized tubes and were left to clot at room temperature. Then, the samples were centrifuged for 20 min at 4,000 rpm to obtain serum and stored at −20°C for biochemical analysis. The animals were euthanized by cervical dislocation, and the heart of each rat was excised and rinsed with cold PBS; then, a small section of the heart was homogenized with an electric homogenizer in cold saline (1: 10, w/v) on ice. Homogenates were then centrifuged at 4°C for 10 min at 12,000 rpm, and the supernatants were preserved at −20°C for further analysis. Another slice of the cardiac tissue was fixed in 10% buffered formalin for histopathological examination.

### 2.5 Estimation of cardiac injury markers

Creatine kinase myocardial band (CK-MB) and lactate dehydrogenase (LDH) serum levels and other toxicity markers, including ALT, AST, and ALP activities, were estimated spectrophotometrically by HumaReader HS (Human, Germany) using available commercial kits (Linear Chemicals, Barcelona, Spain).

### 2.6 Assessment of oxidative stress and inflammatory markers

The supernatant obtained from homogenized heart tissue was used to assess the oxidative/antioxidant status by measuring malondialdehyde (MDA), glutathione (GSH), superoxide dismutase (SOD), catalase (CAT), and glutathione peroxidase (GPx) levels; in addition, inflammatory biomarkers including interleukin-1β (IL-1β) and tumor necrosis factor α (TNF-α) levels were determined using commercially available ELISA kits obtained from MyBioSource (San Diego, United States).

### 2.7 Assessment of cardiac apoptosis markers

The activity of caspase 3 and Bax was assessed using ELISA kits obtained from MyBioSource (San Diego, United States), in accordance with the manufacturer’s instructions.

### 2.8 Real-time quantitative reverse transcription polymerase chain reaction analysis

Following the manufacturer’s protocol, total RNA was extracted from heart tissues using the TransZol Up Plus RNA Kit (TransGen Biotech, China). The purity of RNA was determined by 260/280 ratio using NanoDrop 2000c (Thermo Fisher Scientific, United States). cDNA was synthesized using EasyScript^®^ One-Step gDNA Removal and cDNA Synthesis SuperMix Kit (TransGen, China), and quantification of mRNA using qPCR was carried out utilizing TransStart^®^ Top Green qPCR SuperMix (TransGen Biotech, China) with forward and reverse primers on rotor gene Q, Qiagen. The relative changes in mRNA expression normalized to the GAPDH level (housekeeping gene) for every gene were calculated using the ΔΔCt method (2^−ΔΔCT^), also known as the “Livak method”. Primer sequences were designed using “Primer Quest” (IDT, United States); the primer sequences for glyceraldehyde-3-phosphate dehydrogenase (GAPDH), nuclear factor erythroid 2-related factor 2 (Nrf2), heme oxygenase-1 (HO-1), NAD(P)H: quinone oxidoreductase (NQO-1), Kelch-like ECH-associated protein 1 (Keap1), and nuclear factor kappa B (NF-κB) are shown in Supplementary Table S1. Primers were purchased from Alpha DNA, Canada.

### 2.9 Western blot analysis

The heart tissues were washed with cold PBS and lysed using a strong RIPA lysis buffer in the presence of protease phosphatase inhibitor cocktail (cat. no. E-BC-R327; Elabscience Technologies, Inc.). The protein concentration was determined using the BCA Protein Assay Kit (cat. no. E-BC-K318; Elabscience Technologies, Inc.). Aliquots of 30 μg of protein from each sample were separated by 10% SDS–polyacrylamide gel electrophoresis and transferred onto a PVDF membrane (Bio-Rad, United States). After blocking with 5% skim milk for 90 min, membranes were incubated with specific antibodies, namely, anti-Nrf2 polyclonal antibody (MyBioSource: MBS8001991, 1:1,000), anti-HO-1 (MyBioSource: MBS2538072, 1:1,000), and B-actin (Elabscience: E-AB-40517, 1:1,000) overnight at 4°C, followed by incubation with the secondary antibody, namely, HRP-conjugated anti-rabbit IgG at 1:1,000 dilution (Elabscience: E-AB-1003) for 1 h at room temperature. Antibody binding with the protein of interest was detected with chemiluminescence (ECL, Bio-Rad, United States). Quantification was performed by densitometry. The results were normalized to beta-actin.

### 2.10 Histopathological cardiac damage assessment

Fixed 10% formalin heart tissues were dehydrated with ethanol and embedded in paraffin; the tissues were cut into ∼5 μm thick sections, then stained with hematoxylin and eosin (H&E) stain and examined under a light microscopic. The images were scored semiquantitatively depending on the extent of necrosis of the cardiac tissue and inflammatory cell infiltration following the scoring scale: score 0, normal; score 1, damage up to 25%; score 2, between 25% and 50%; score 3, between 50% and 75%; and score 4, above 75% (Kanda et al., 2004); the examination was carried out by a senior pathologist, blinded to the study.

### 2.11 *In situ* cell death (apoptosis)/TdT-mediated dUTP-biotin nick end labeling assay

Cellular apoptosis in cardiac tissue was determined at the nuclear level using the One-Step TUNEL *In Situ* Apoptosis Kit (green, FITC) (Cat No: E-CK-A320, Elabscience, United States), in accordance with the manufacturer’s protocol. TUNEL-positive cells were detected under a fluorescence microscope, and photomicrographs were taken at ×1000 magnification. For TUNEL quantification, seven selected fields in each slide were examined, and the mean and the percentage were calculated; the examination was carried out by a senior pathologist who was blinded to the study. The apoptosis indicator (apoptotic index) was estimated as the fraction (%) of the labeled nuclei to the total number of nuclei counted.

### 2.12 Statistical analysis

The data of this study were demonstrated as mean ± standard deviation (SD), RT-qPCR data were expressed as mean ± standard error of mean (SEM), and the statistical significance among groups was determined using the one-way analysis of variance (ANOVA) test followed by Tukey’s *post hoc* test for multiple comparisons between groups using GraphPad Prism software (version 9.5.0). *, *p* < 0.05; **, *p* < 0.01; ****, *p* < 0.0001; ###, *p* < 0.001; and ####, *p* < 0.0001 values across all figures were statistically significant.

## 3 Results

### 3.1 Effect of cafestol on cardiac injury marker levels and histopathological changes

**FIGURE 1:**
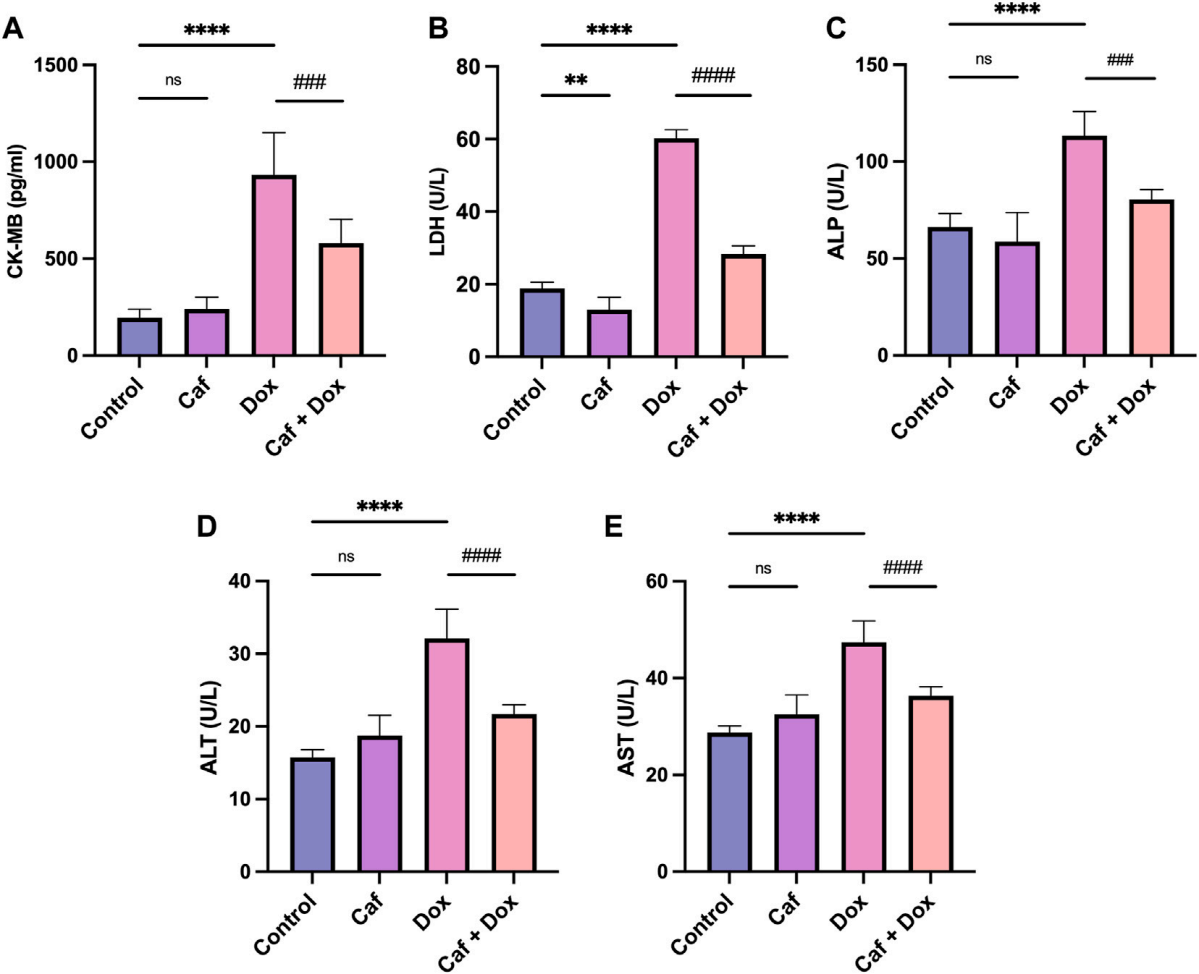
Effect of cafestol on cardiac tissue injury markers in Wister rats. **(A)** Effect of cafestol on CK-MB serum levels. **(B)** Effect of cafestol on LDH serum levels. **(C)** Effect of cafestol on ALP serum levels. **(D)** Effect of cafestol on ALT serum levels. **(E)** Effect of cafestol on AST serum levels. Data are expressed as mean ± SD, *n* = 8. **, *p* < 0.01; ****, *p* < 0.0001 vs. control group; ###, *p* < 0.001; ####, *p* < 0.0001 vs. DOX group; ns (*p* > 0.05), no significant difference.

Figure 1 shows that IP-administered DOX alone (group III) caused cardiac injury, as seen in the significant (*p* < 0.0001) elevated serum levels of CK-MB, LDH, AST, ALT, and ALP compared to the control group (group I). However, pre-treatment with cafestol at 5 mg/kg b.w. prior to DOX exposure (group IV) showed a significant reduction in the measured parameters’ serum levels compared to the DOX-only group, which indicated that cafestol has a protective role against DOX-induced cardiac injury.

**FIGURE 2:**
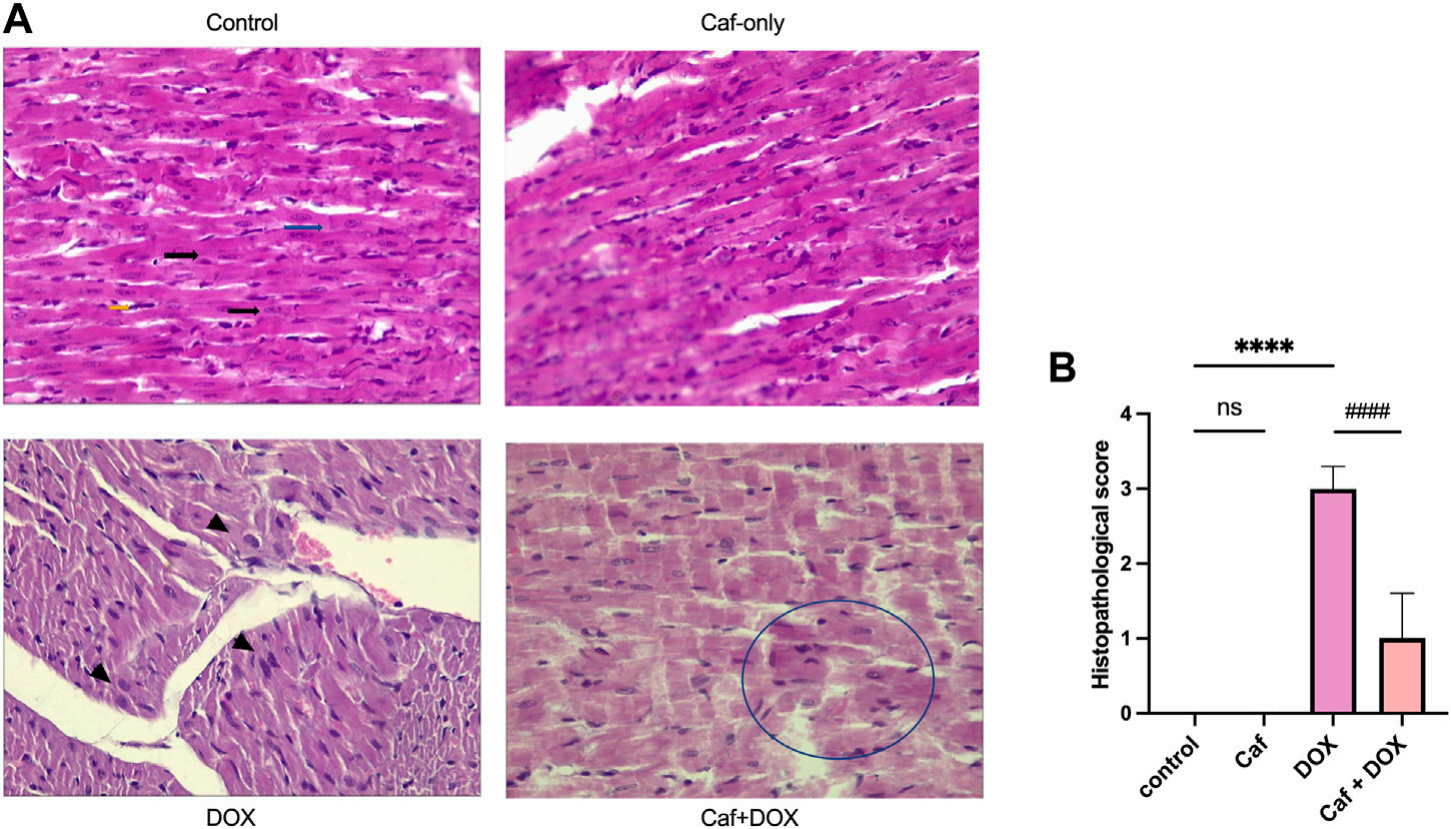
Effect of cafestol on cardiac tissue histopathological change in Wister rats. **(A)** Photomicrographs showing the pathological morphology of myocardial tissue (H&E ×400); the control group has normal histology of longitudinally oriented cardio-myofibers from the left ventricle of the rat, with large oval nuclei containing granular chromatin (black arrows) and with faint intercalated discs that are also well demonstrated (blue arrows). The typical branched appearance of the cardiac muscle is also well preserved (yellow arrows). The DOX group showed dilated cardiomyopathy and myocardial necrosis within the longitudinal section in a rat’s heart, manifested as areas of pallor in the myocardium with loss of cellular details, and myocardium coalesces, with large nuclei starting to appear (arrowhead). In the caf group, cardiac tissue myofibers are well preserved and appeared with normal architecture. In the caf + DOX group, cardiac tissue general architecture of the left ventricle showed reduced signs of myocardial damage with some losses of cellular architecture in the form of “merged” myofibers (encircled area). **(B)** Semiquantitative analysis of the histological score in the rat myocardium following the scale: score 0, normal; score 1, lesion up to 25%; score 2, between 25% and 50%; score 3, between 50% and 75%; and score 4, above 75%; seven selected fields in each slide were examined for necrosis and inflammatory cells infiltration. Data are expressed as mean ± SD, *n* = 8. ****, *p* < 0.0001 vs. control group; ####, *p* < 0.0001 vs. DOX group; ns (*p* > 0.05), no significant difference.

Consistently, in Figures 2A, B, the rats’ heart tissues exposed to DOX alone (group III) showed a significantly higher (*p* < 0.05) cardiac pathological score as characterized by the myocardial tissue architectural disturbance (fragmentation and degenerated wavy myofibers with absent striations), necrosis, and inflammatory cell infiltration than the control group (group I), which showed normal myocardial structure (branching and anastomosing cardiac myofibers) with no necrosis or infiltration with inflammatory cells. However, pre-treatment with cafestol (group IV) maintained cardiac myofiber organization with less myofibrillar loss (Figure 2A); additionally, cafestol pre-treatment at 5 mg/kg b.w. showed significantly (*p* < 0.05) lower cardiac semiquantitative injury scores than group III as demonstrated in Figure 2B. Notably, cafestol alone (group II) showed comparable changes in the levels of cardiac injury markers, as exhibited by the control group (group I), with no significant difference (Figure 1). In addition, cafestol alone did not cause histopathological changes in the cardiac tissue with an appearance comparable to that of the control group (Figure 2A), with a cardiac pathological score similar to that exhibited by the control group (Figure 2B), indicating that cafestol at 5 mg/kg b.w. is safe and has no toxic effect.

### 3.2 Effect of cafestol on cardiac oxidative stress

**FIGURE 3:**
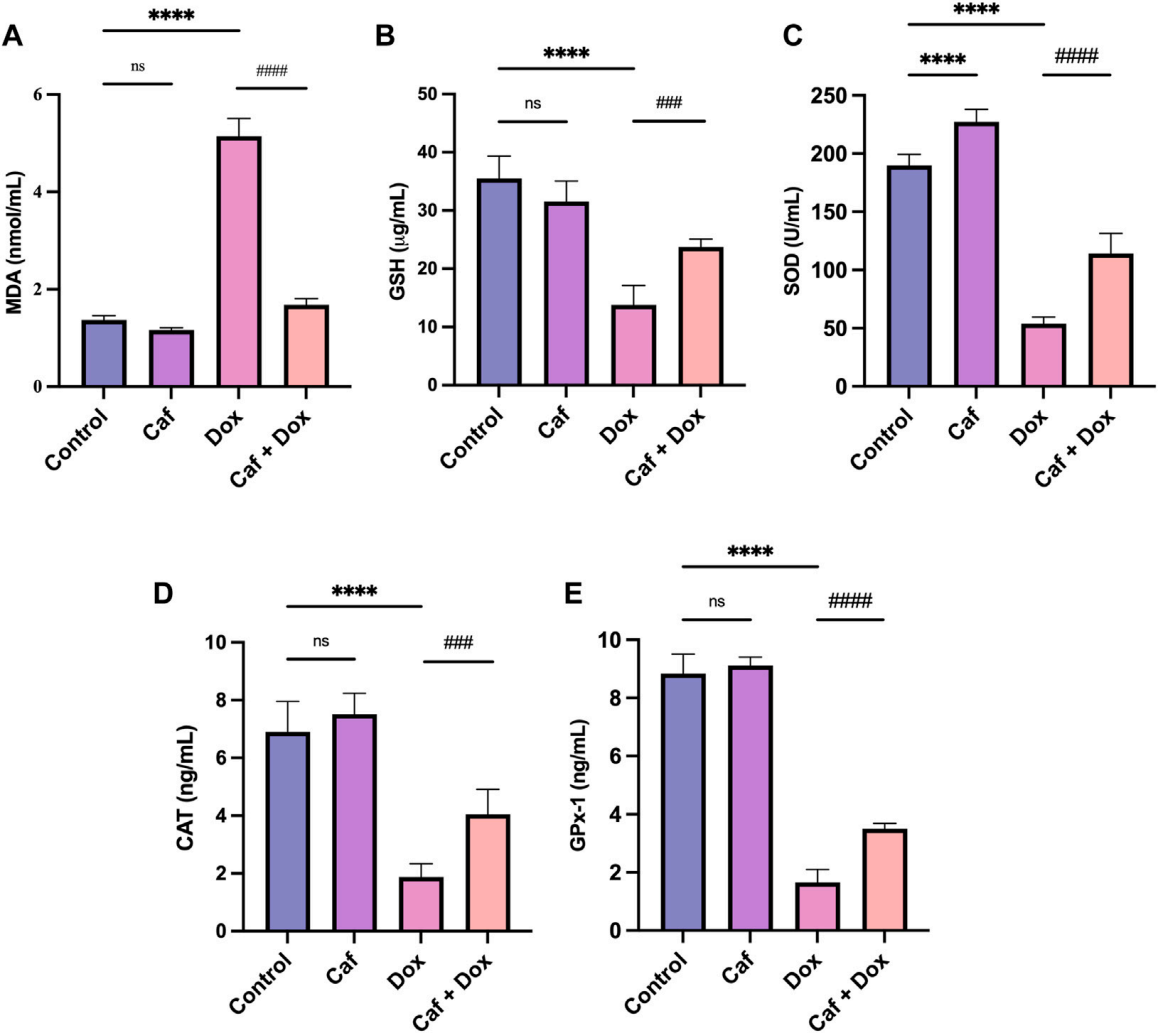
Effect of cafestol on cardiac tissue oxidative stress markers in Wister rats. **(A)** Effect of cafestol on MDA. **(B)** Effect of cafestol on GSH. **(C)** Effect of cafestol on SOD. **(D)** Effect of cafestol on CAT. **(E)** Effect of cafestol on GPx-1. Data are expressed as mean ± SD, *n* = 8. ****, *p* < 0.0001 vs. control group; ###, *p* < 0.001; ####, *p* < 0.0001 vs. DOX group; ns (*p* > 0.05), no significant difference.

The cardiac antioxidant activity of cafestol was assessed in rats following acute DOX exposure, as oxidative stress is important in DOX-mediated myocardial damage. Administration of DOX alone (group III) caused a highly significant increase (*p* < 0.0001) in the MDA level compared to the control group (group I); however, pre-treatment with cafestol (group IV) significantly ameliorated the increase in the MDA level (Figure 3A). On the other hand, DOX alone (group III) caused a significant GSH depletion and SOD, CAT, and GPx-1 exhaustion compared to the control group (group I), while pre-treatment with cafestol (group IV) significantly increased the levels of GSH and restored SOD, CAT, and GPx-1 activity in comparison to the DOX group (group III) (Figures 3B–E). Furthermore, cafestol alone (group II) caused no significant difference in the MDA level (*p* > 0.05) Figure 3A and significantly increased the SOD tissue level when compared to the control group (group I) Figure 3C.

### 3.3 Effect of cafestol on cardiac inflammatory marker levels and NF-κB gene expression

**FIGURE 4:**
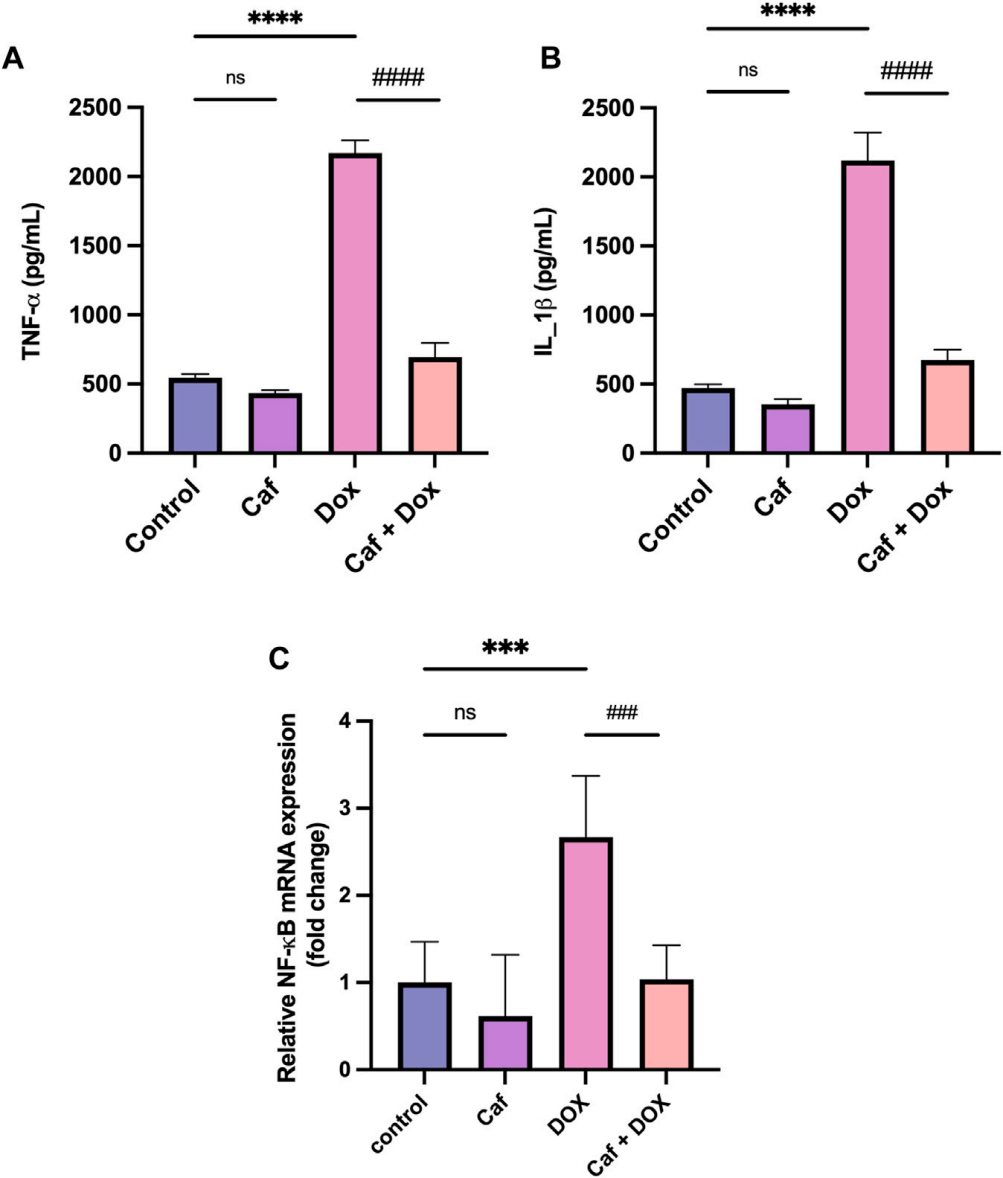
Effect of cafestol on cardiac tissue inflammatory markers in Wister rats. **(A)** Effect of cafestol on TNF-α. **(B)** Effect of cafestol on IL-1β. Data are expressed as mean ± SD, *n* = 8. **(C)** Effect of cafestol on NF-κB. Data are expressed as mean ± SEM, *n* = 8. ***, *p* < 0.001; ****, *p* < 0.0001 vs. control group; ###, *p* < 0.001; ####, *p* < 0.0001 vs. DOX group; ns (*p* > 0.05), no significant difference.

Inflammation is one of the leading triggers of cardiomyocyte death and the subsequent cardiac dysfunction; for this, the proinflammatory cytokines’ (IL-1β and TNF-α) cardiac tissue levels and NF-κB gene expression were analyzed. Figures 4A, B show that there was a significant increase (*p* < 0.0001) in the cardiac tissue level of TNF-α and IL-1β in the DOX group compared to the normal control group. Furthermore, the expression of the NF-κB gene in the cardiac tissue significantly increased (*p* < 0.001) in the model group (group III), as shown in Figure 4C. However, cafestol pre-treatment in group IV significantly reduced cardiac tissue levels of TNF-α and IL-1β and inhibited the expression of the NF-κB gene compared to the model group (group III). Cafestol alone (group II) showed comparable cardiac levels of TNF-α, IL-1β, and NF-κB gene expression to those of the normal control group (group I).

### 3.4 Effect of cafestol on cardiomyocyte apoptosis

**FIGURE 5:**
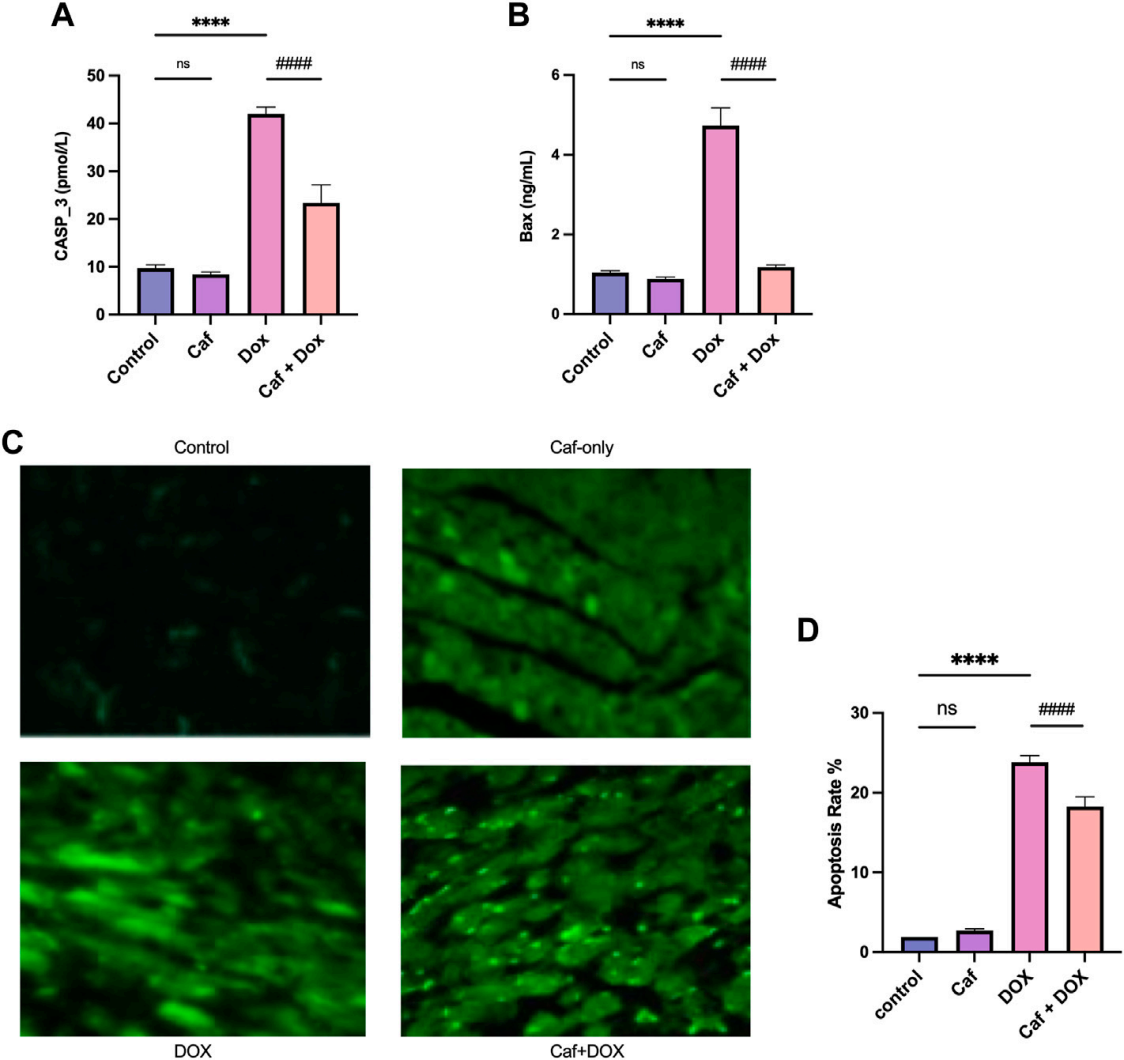
Effect of cafestol on cardiac tissue apoptosis in Wister rats. **(A)** Effect of cafestol on Casp3 cardiac tissue levels. **(B)** Effect of cafestol on Bax cardiac tissue levels. **(C)** Photomicrographs showing TUNEL-positive cardiomyocytes (hyperchromatic-bright green), ×1,000 magnification. **(D)** Analysis of TUNEL-positive cells quantitatively in the rat myocardium. Data are expressed as mean ± SD, *n* = 8. ****, *p* < 0.0001 vs. control group; ####, *p* < 0.0001 vs. DOX group; ns (*p* > 0.05), no significant difference.

Apoptotic cell death of the cardiomyocytes is a causative factor of heart failure following the use of doxorubicin; thus, we examined the cardiac tissue levels of both caspase 3 and Bax. As shown in Figures 5A, B, there was a significant increase (*p* < 0.0001) in the cardiac tissue levels of both caspase 3 and Bax in the model group (group III) compared to the control group (group I); however, pre-treatment with cafestol prior to acute exposure to DOX (group IV) induced a significant inhibition in the apoptotic markers compared to the model group, while cafestol alone (group II) has no significant effect on Casp 3 and Bax cardiac tissue levels compared to the control group.

On the other hand, the evaluation of apoptotic cells at the nuclear level using the TUNEL assay (TUNEL-positive cardiomyocytes) (Figure 5C) showed that acute exposure to DOX (group III) induced an increase in the appearance of TUNEL-positive cardiomyocytes in the form of hyperchromatic patches of bright green (indicating DNA fragmentation along adjacent rows of cells) with a significantly higher (*p* < 0.0001) apoptosis rate (Figure 5D) than the control group. However, pre-treatment with cafestol reduced the number of TUNEL-positive cardiomyocytes (seen as scattered apoptotic cells) and significantly reduced the apoptosis rate compared to the model group. However, cafestol alone (group II) showed a TUNEL-positive cardiomyocyte appearance comparable to that of the control group with no significant difference (*p* > 0.05) in the apoptotic index in comparison to the control group.

### 3.5 Effect of cafestol on the Nrf2 pathway and its downstream antioxidant genes

**FIGURE 6:**
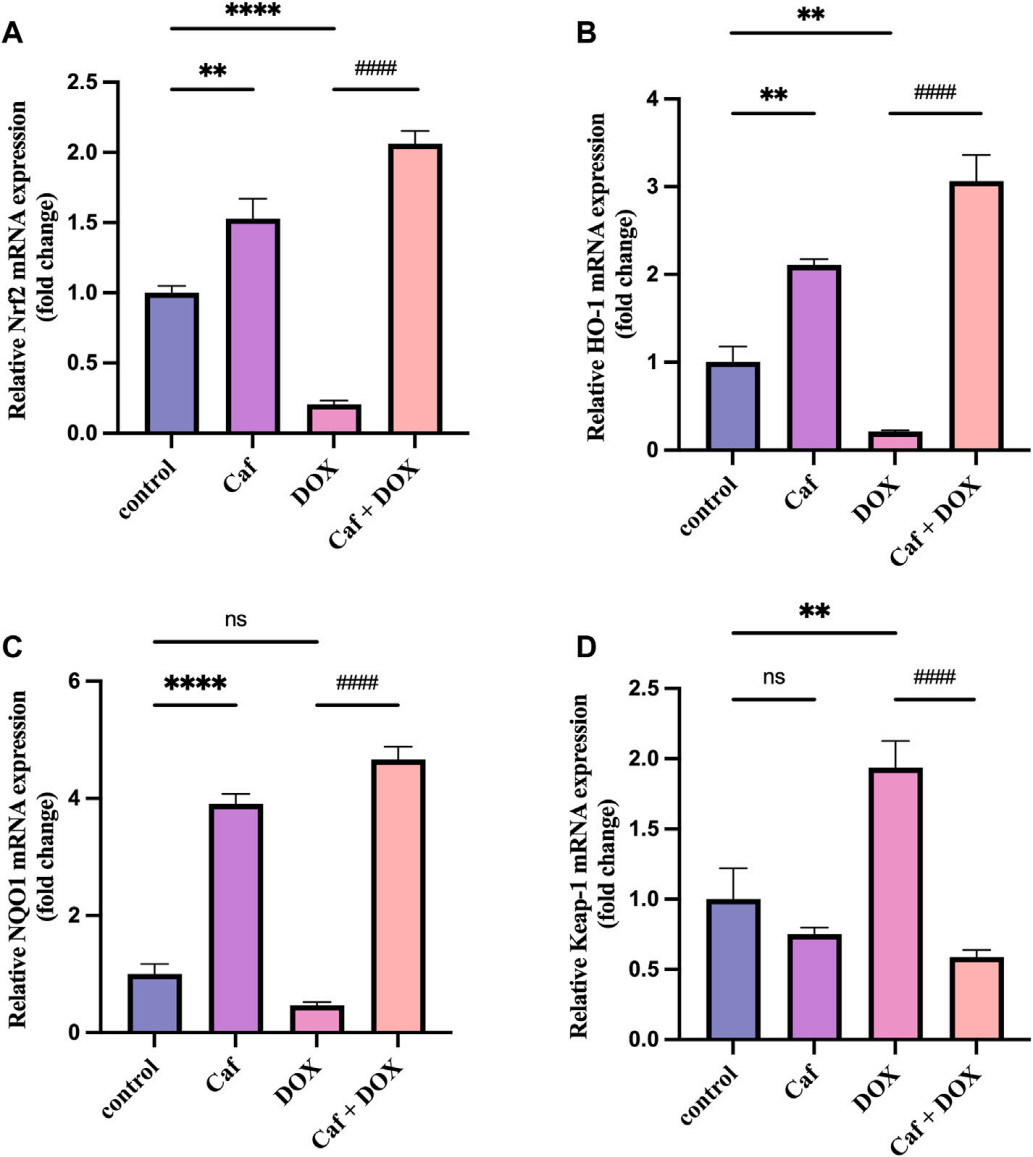
Effect of cafestol on the Nrf2/Keap1 pathway. **(A)** Effect of cafestol on Nrf2 gene expression. **(B)** Effect of cafestol on HO-1 gene expression. **(C)** Effect of cafestol on NQO-1 gene expression. **(D)** Effect of cafestol on Keap1 gene expression. Data are expressed as mean ± SEM, *n* = 8. **, *p* < 0.01; ****, *p* < 0.0001 vs. control group; ####, *p* < 0.0001 vs. DOX group; ns (*p* > 0.05), no significant difference.

To explain the mechanisms underlying the cardioprotective effects of cafestol pre-treatment, we assessed the effects of cafestol on Nrf2, Keap1, HO-1, and NQO-1 gene expression in myocardial tissue. Figure 6 shows that the Nrf2 and HO-1 genes’ expression were significantly decreased (*p* < 0.0001) and (*p* < 0.01), respectively, and there was a significant increase (*p* < 0.01) in the expression of the Keap1 gene in the model group (group III) compared to group I. In addition, doxorubicin alone caused a non-significant change in the NQO-1 gene expression in comparison to group I (control group).

However, the mRNA expression levels of Nrf2, HO-1, and NQO-1 in group IV (cafestol pre-treatment + DOX) were significantly higher (*p* < 0.0001) than those in group III (doxorubicin only); in addition, cafestol pre-treatment significantly reduced (*p* < 0.0001) the cardiac tissue gene expression of Keap1 compared to the model group. On the other hand, cafestol alone (group II) significantly increased cardiac tissue mRNA levels of Nrf2, HO-1, and NQO-1 with no significant effect on Keap1 levels compared to the control group.

**FIGURE 7:**
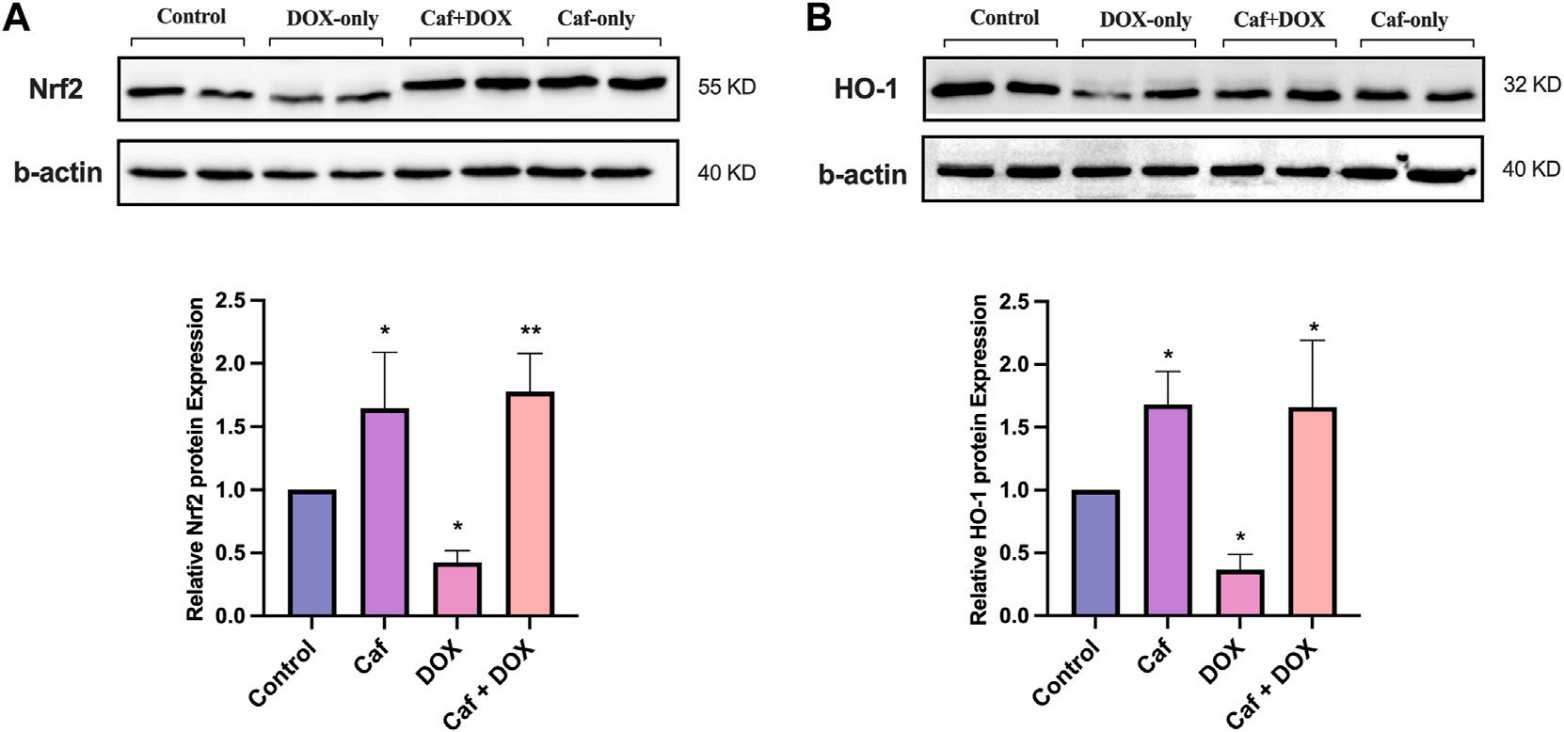
Effect of cafestol on cardiac tissue Nrf2 and HO-1 protein expression in Wister rats. **(A)** Representative western blot bands and the relative quantification of Nrf2 protein expression. **(B)** Representative western blot bands and the relative quantification of the level of HO-1 protein level. Data are expressed as mean of fold change relative to the control ±SD; *n* = 4. *, *p* < 0.05; **, *p* < 0.01 vs. control group.

In addition, the effect of cafestol on Nrf2 protein expression was examined in the rats’ heart tissue; Figure 7A reveals that group III (acute exposure to doxorubicin) exhibited a significantly reduced (*p* < 0.05) Nrf2 protein expression; in contrast, cafestol pre-treatment prior to DOX exposure (group IV) significantly increased (*p* < 0.01) the Nrf2 protein expression in the heart tissue. Moreover, the administration of cafestol alone (group II) increased the cardiac tissue Nrf2 protein expression significantly (*p* < 0.05).

For cardiac tissue HO-1 protein levels, as demonstrated in Figure 7B, the exposure to doxorubicin (group III) caused a significant reduction (*p* < 0.05) in HO-1 protein expression; while in group IV, the HO-1 protein expression in the heart tissue showed a significant increase. Furthermore, in group II, there was a significant increase in HO-1 protein expression.

## 4 Discussion

Although DOX is used for treating a broad range of solid tumors, its use is associated with severe cardiotoxicity, including arrhythmia, reduced ejection fraction, cardiomyopathies, and heart failure (Skovgaard, Hasbak, and Kjaer, 2014). The mechanism that mediates doxorubicin-induced cardiotoxicity is unclear; however, it might be related to oxidative stress, inflammatory cascade, apoptosis, and DNA damage; among them, oxidative stress plays an important role in DOX-induced toxicities through the generation of free radicals that cause depletion of antioxidants, increasing lipid, protein, and nucleic acid peroxidation and disturbing mitochondrial function (Rocca et al., 2020) (Ridha and Nada, 2019).

As Nrf2 plays a critical role in regulating oxidative stress within cells, once oxidative stress occurs, Nrf2 translocates into the nucleus after dissociating from Keap1 due to the oxidation of the active site of Keap1, triggering the expression of different antioxidant genes; in addition, Nrf2 signaling pathway activation can efficiently preserve cellular redox homeostasis and modulate apoptotic protein levels with an efficient anti-inflammatory function, which helps alleviate myocardial infarction and other cardiovascular disorders (Wu et al., 2022) (Saha et al., 2020).

In the current study, the data showed that acute exposure to DOX produced cardiac injury, represented by the change in the histological appearance of cardiac tissue and the significant increase in serum levels of CK and LDH as indicators of clinical myocardial injury; in addition, DOX caused oxidative stress as seen in the elevated cardiac tissue level of MDA, depleted GSH and antioxidant enzymes, and induced the apoptosis and inflammatory cascade by increasing the Bax and caspase 3 and TNF-α and IL-1β cardiac tissue levels. Furthermore, DOX alone suppressed the Nrf2 pathway by enhancing Keap1 gene expression and reducing Nrf2 and HO-1 mRNA tissue levels compared to group I (control group). These results are agreeable with previous studies affirming that DOX caused cardiac damage and oxidative stress and induced apoptosis and inflammation (Dundar et al., 2016) (Sirwi et al., 2022) (Zhou et al., 2022) (Ridha and Nada, 2019); thus, DOX-induced cardiotoxicity was successfully established.

Our result revealed that cafestol (5 mg/kg/day) alone caused a non-significant difference in CK and LDH serum levels with a comparable cardiac tissue histological appearance to that of the control group; this indicates that cafestol alone has no cardiotoxic effect on rats’ heart *in vivo*. However, cafestol pretreatment efficiently protected against doxorubicin-induced cardiac injury as it significantly reduced the serum levels of CK and LDH and other cellular injury biomarkers; in addition, cafestol ameliorated the histopathological changes and significantly reduced the apoptosis and inflammatory markers. Furthermore, cafestol pre-treatment significantly increased the cardiac tissue levels of GSH, SOD, CAT, and GPx-1, reduced MDA tissue levels, and promoted Nrf2 and HO-1 proteins and gene expression and NQO-1 gene expression; in addition, cafestol significantly decreased the expression of the Keap1 gene, thus inhibiting the oxidative stress-mediated damage in cardiomyocytes when compared to animals treated with doxorubicin alone; therefore, cafestol might have a beneficial role in alleviating doxorubicin-mediated acute cardiac injury.

The mechanism that may explain the reduction of doxorubicin-induced cardiotoxicity might be related to the cafestol protective effect as a result of its antioxidant activity through the activation of the Nrf2 pathway, as it may have reduced doxorubicin-mediated free radicals’ generation since doxorubicin is well recognized to induce cellular oxidative stress. Studies showed that cafestol, a coffee-specific diterpene, has antioxidant and anticarcinogenic effects in animal models as a result of modulating the Nrf2 axis (Lima et al., 2017) (Cavin et al., 2002) (Miller et al., 1991), which is related to cafestol’s ability to enhance the expression of glutathione-S-transferases (GSTs) (Lam, Sparnins, and Wattenberg, 1982). Cafestol in a mixture with kahweol was previously reported to prevent aflatoxin B1-induced genotoxicity through a dual mechanism that involved the modulation of xenobiotic-activating enzymes that have the ability to activate potential carcinogens and increase the expression of GST that, in turn, provides a chemoprotective effect (Cavin et al., 1998). Furthermore, cafestol activates the Nrf2/HO-1 pathway, thus inhibiting the redox signaling (Hao et al., 2019). Furthermore, cafestol has been reported to interfere with NF-κB-dependent transcriptional activity, thus reducing inflammatory-mediated cardiac damage induced by doxorubicin (Kim, Jung, and Jeong, 2004) (Kathem, Abdulsahib, and Zalzala, 2022). Similarly, cafestol has been found to attenuate apoptosis and protect cells against oxidative stress and DNA damage induced by hydrogen peroxide (Ji et al., 2020) (Lee and Jeong, 2007). The results of the present study indicated that the protective effect of cafestol against DOX-induced cardiotoxicity depended on the activation of the Nrf2 signaling pathway.

## 5 Conclusion

According to results obtained from this study, it can be concluded that oral administration of cafestol (5 mg/kg/day) alone has no cardiotoxic effect; cafestol pre-treatment mitigated the DOX-induced cardiotoxicity and myocardial injury, inhibited apoptosis, and ameliorated oxidative stress and inflammatory response through the activation of the Nrf2 signaling pathway. Cafestol might be a potential chemoprotective agent against doxorubicin-induced adverse effect in cancer chemotherapy. However, additional *in vivo* and *in vitro* experiments on the antioxidant and antiapoptotic roles of cafestol on the cardiotoxicity model induced by doxorubicin, further investigation of the mechanisms involved, and the evaluation of other routes of administration and different doses, in addition to comparing the efficacy of effect of cafestol to other cardioprotective agents, are required.

## Data Availability

The original contributions presented in the study are included in the article/Supplementary Material; further inquiries can be directed to the corresponding author.
