# Supplementary material for: Protective effect of cafestol against doxorubicin-induced cardiotoxicity in rats by activating the Nrf2 pathway
Source: Front Pharmacol. 2023 Jun 12;14:1206782. doi: 10.3389/fphar.2023.1206782 (PMC10291064; doi:10.3389/fphar.2023.1206782)
Supplement: Supplementary file 1 [file Table1.DOCX]

Table S1. Oligonucleotide sequences of primers used for RT-qPCR.

| Gene name | Forward (5′ -3′) | Reverse (5′ -3′) |
| --- | --- | --- |
| Nrf2 | GCTATTTTCCATTCCCGAGTTAC | ATTGCTGTCCATCTCTGTCAG |
| HO-1 | CTTTCAGAAGGGTCAGGTGTC | TGCTTGTTTCGCTCTATCTCC |
| NQO1 | CTGGCCAATTCAGAGTGGCAT | GAGTGGTGACTCCTCCCAGA |
| Keap1 | AATGCTATGACCCAGACACTG | CAGGTAGTCCAAGTGCTTCAG |
| NF-κB | AAGCAGGAAGATGTGGTGGA | GATAAGGAGTGCTGCCTTGC |
| GAPDH | TTCCAGGAGCGAGATCCCGCTAAC | CATGAGCCCTTCCACGATGCCAAAG |
